# Dietary diversity and development among early childhood children in rural China

**DOI:** 10.3389/fpubh.2024.1485548

**Published:** 2024-12-02

**Authors:** Yanyan Qin, Ai Yue, Yali Zhang, Xinyue Zhang, Yuan Gao, Shibin Liang, Qiannan Song, Na Qiao

**Affiliations:** ^1^Center of Experimental Economics in Education, Shaanxi Normal University, Xi'an, Shaanxi, China; ^2^Faculty of Education, Shaanxi Normal University, Xi'an, Shaanxi, China; ^3^College of Preschool Education, Xi'an University, Xi'an, Shaanxi, China

**Keywords:** dietary diversity, cognitive development, non-cognitive development, health, rural China

## Abstract

**Background:**

Early childhood is a critical period for cognitive development, heavily influenced by nutrition. Despite significant economic advancements, malnutrition and micronutrient deficiencies persist in many low- and middle-income countries, including China, where dietary diversity remains suboptimal. Existing research predominantly relies on correlational data, underscoring the need for more rigorous empirical evidence. This study aims to fill that gap by providing stronger empirical evidence on the relationship between dietary diversity and developmental outcomes in rural Chinese children.

**Methods:**

We conducted a longitudinal cohort study of 1,207 children aged 6–23 months, drawn from 100 villages across 22 counties in rural China, with three follow-up rounds extending until the children reached 51–83 months of age. Cognitive and non-cognitive development, as well as key health indicators, were evaluated. Cognitive development was measured using the Bayley Scales of Infant and Toddler Development and the Wechsler Preschool and Primary Scale of Intelligence Fourth Edition. Non-cognitive development was assessed via the Ages and Stages Questionnaire: Social-Emotional and the Strengths and Difficulties Questionnaire. Health indicators included anemia, frequency of illness in the past 2 weeks, and four key anthropometric measures (height-for-age *z*-scores, weight-for-age *z*-scores, weight-for-height *z*-scores, and body mass index-for-age *z*-scores). Multiple linear regression models were applied to analyze the relationship between dietary diversity scores (DDS) and developmental outcomes, incorporating child fixed effects and adjustments for time-variant confounders. We accounted for the hierarchical structure of the data by clustering standard errors at the village level, which also reflects the township level.

**Results:**

The regression analysis identified significant positive associations between DDS and cognitive (*p* < 0.01), as well as reduced illness incidence (*p* < 0.001) in children aged 6–54 months. Additionally, DDS demonstrated a strong positive effect on non-cognitive development (*p* < 0.001) in children 2 years and older. The associations between DDS, non-cognitive development (*p* < 0.01), and illnesses reduction (*p* < 0.001) remained robust under two-way fixed effects models.

**Implications:**

To promote sustainable improvements in child development and health, policymakers should prioritize nutrition-focused interventions in rural areas. Community-based programs offering caregivers training and nutritional support, integrated within healthcare and social services infrastructures, are vital for ensuring families have the necessary resources enhance child wellbeing and long-term developmental outcomes.

## 1 Introduction

The first 5 years post-birth are crucial for shaping children's cognitive functions, characterized by rapid brain growth and neural plasticity (1, 2). While genetics factors lay the foundation for developmental potential, external influences such as nutrition play a critical role in determining whether this potential is fully realized (3). Insufficient nutrition during infancy and early childhood may lead to micronutrient deficiencies, impair immune function, and have enduring consequences on growth, development, human capital accumulation, with repercussions that may extend into adult earnings and economic productivity (4).

Malnutrition remains a major challenge in many low- and middle-income countries, contributing to 45% of global deaths in children under five (5). In China, despite its status as the world's second-largest economy and a leading agricultural nation, the problem persists. The prevalence of severe anemia among infants aged 6–12 months in rural areas stands at 48.3%, and although it decreases after 18 months, it remains high at 23.3% for children aged 24–30 months (6). This nutrition deficiency not only impedes health and development but also undermines China's broader efforts to make substantial contributions to global development goals.

Suboptimal nutritional outcomes are often linked to poor complementary feeding practices, emphasizing the importance of dietary diversity (7). Food and Agriculture Organization (FAO) recognizes dietary diversity as a key predictor of diet quality and a reliable indicator of micronutrient adequacy, especially for young children (8). The World Health Organization (WHO) recommends that children aged 6–23 months consume a diverse diet comprising five or more food groups daily to ensure sufficient micronutrient intake (9).

Globally, however, most children fall short of this dietary diversity standard. Over two-thirds of children aged 6–23 months (69%) do not receive an adequately varied diet during early childhood (10). This problem is particularly pronounced in low- and middle-income countries, where children's diets often rely heavily on starchy staples, with limited access to animal products and vitamin A-rich fruits and vegetables (11, 12). Several factors, including children's age, maternal education, household wealth, geographical location, are strongly associated with dietary diversity (13, 14). Research consistently show that children of higher-educated mothers and those from wealthier households have greater access to a diverse range of foods (15).

A diverse diet has been shown to enhance physical wellbeing (1), reduce illness (16), and support overall child development. A longitudinal study tracking 117 children aged 6–23 months over a 6-month period found that greater dietary diversity was associated with significant improvements in both cognitive and non-cognitive development, as well as growth, without showing differences across various subgroups (17). However, findings in this area are not unanimous. For instance, a large cross-sectional study by Bliznashka et al., which included 12,126 children aged 36–59 months across 15 low- and middle-income countries, found no significant association between dietary diversity and cognitive, socio-emotional, or physical development (18).

Despite the substantial body of research on dietary diversity in low- and middle-income countries, there remains a paucity of evidence from rural China. This gap is notable given the ongoing high level of undernutrition among children in these areas. Existing studies in this context predominantly employ a correlational approach. For example, Li et al. reported a positive correlation between dietary diversity and cognitive performance based on cross-sectional data from 1,334 children aged 3–5 years in rural Hunan Province (19). Similarly, Zhao et al. found an association between minimum dietary diversity and reduced developmental delays using cross-sectional survey data from 1,534 infants aged 6–23 months in six rural Chinese counties (20). While these studies shed light on important health-related outcomes (21–23), they often lack the depth needed to fully explore the multidimensional relationship between dietary diversity and child development, particularly cognition and non-cognition.

This study aims to fill these gaps by utilizing panel data to explore the relationship between dietary diversity and cognitive, non-cognitive, and health outcomes in rural China. We first document the dietary diversity patterns among children aged 6–83 months and then explore the associations between dietary diversity and various dimensions of child development.

## 2 Methods

### 2.1 Study design

This study was conducted in 100 villages across 22 counties in the rural Qinba Mountain area of northwest China. The per capita disposable income of rural residents in the sample area was $1,395 (RMB 8,689) in 2015, lower than the national average of $1,834 (RMB 11,422) for rural residents in national poverty-stricken areas (24).

A multistage cluster sampling design was employed to select children at the household level. First, 20 nationally designated poverty counties (classified as “poverty counties” in 2015) were randomly selected from three cities in the Qinba areas. Since counties are smaller administrative units governed by city administrations, this allowed for a geographically diverse sample. Next, 100 townships were selected from the forementioned 22 counties, excluding townships located at the county centers (considering their tendency to be wealthier and more urban), and townships without any villages with population of at least 800 people. One village was then randomly selected from each township, resulting in a total of 100 surveyed villages. The baseline survey included 1,709 children and their families.

Following the baseline survey in 2015, three follow-up surveys were conducted until 2021, covering children at four different age intervals: 6–23, 16–42, 29–54, and 51–83 months. Missing data for dietary diversity, child development, and control variables from 2015 to 2021 were excluded from the analysis. Ultimately, 1,207 children and their caregivers participated in the baseline survey (wave 0), 1,654 in the first follow-up (wave 1), 1,248 in the second follow-up (wave 2), and 1,350 in the third follow-up (wave 3; Table 1). It is important to note that the baseline survey initially included 1,709 children aged 6–23 months. However, due to incomplete data on breastfeeding practices (one key variable), the final baseline sample size was adjusted to 1,207. Despite this reduction, subsequent follow-up surveys were based on the full original cohort of 1,709 children, leading to larger sample sizes in later waves.

**Table 1:** Frequency or mean ± standard deviation of dietary diversity score at 6–23, 16–42, 29–54, and 51–83 months.

**Dietary diversity score**	**6–23 months**	**16–42 months**	**29–54 months**	**51–83 months**	**Full sample**
Full (0–9)	3.38 ± 1.58	4.71 ± 1.62	4.73 ± 1.65	5.34 ± 1.77	4.58 ± 1.79
Low (0–4; *n* = 2,624)	75.6	45.2	44.7	30.1	48.1
Adequate (5–9; *n* = 2,835)	24.4	54.8	55.3	69.9	51.9
Observations	1,207	1,654	1,248	1,350	5,459

The mean dietary diversity score for children aged 6–23 months is on a scale of 0–8 according to the World Health Organization and United Nations Children's Fund guidelines. Results are given as mean ± standard deviation or as %.

Informed written consent was obtained from all participating parents. We would like to thank all the children and their families who participated in this study. All procedures conducted involving human participants complied with the ethical standards established by the appropriate institutional and/or national research committees, as well as the 1964 Helsinki Declaration and its later amendments or comparable ethical guidelines. The study was approved by the appropriate Biological and Medical Ethics Review Committee under relevant grant provisions.

### 2.2 Data collection

In this study, we utilized three types of collected information: (1) data on children and household characteristics; (2) child development status encompassing cognition, non-cognition, and health; and (3) dietary diversity scores (DDS).

#### 2.2.1 Children and household characteristics

Data on children and household characteristics were systematically collected using a structured questionnaire. Child-level characteristics included the child's age, gender, mode of delivery (natural or otherwise), premature birth status, breastfeeding history, and the presence of siblings. Household-level characteristics comprised the grandmother's health status, mother's age, father's education level, household asset index, government welfare receipt, and the primary caregiver's attributes (i.e., age, educational attainment, and relationship to the child).

#### 2.2.2 Child cognitive development status

Cognitive development was assessed using age-appropriate tools. For children under 42 months, cognitive and language performances were evaluated using the Bayley Scales of Infant and Toddler Development (Bayley-III), widely recognized as the gold standard for early child development assessment (25). The BSID-III has been formally adapted to the Chinese language and environment, demonstrating its suitability for application in numerous studies across rural China (26). For children aged 42 months and older, cognitive and verbal functioning were assessed using the Mandarin language version of the Wechsler Preschool and Primary Scale of Intelligence Fourth Edition (WPPSI-IV). The Chinese adaptation of the WPPSI-IV, tailored to the Chinese language and environment in 2010, has been widely employed in studies conducted throughout China (27, 28). To ensure comparability, all BSID-III and WPPSI-IV scores were normalized to have a mean of zero and a standard deviation of one, considering the child's age in months.

#### 2.2.3 Child non-cognitive development status

Non-cognitive development was assessed using the Ages and Stages Questionnaire: Social-Emotional (ASQ: SE) for children under 66 months old. The ASQ: SE is a widely used tool that evaluates socio-emotional development across skills like self-regulation, compliance, communication, adaptive functioning, autonomy, affect, and interaction with others (29, 30). For ease of interpretation, ASQ: SE scores were reversed, so higher scores indicate better development, while scores below zero suggesting a risk of delays. For children aged 66 months and older, the Strengths and Difficulties Questionnaire (SDQ) was employed (31, 32). This SDQ is behavioral screening tool, assessing five domains: conduct problems, emotional problems, hyperactivity, peer problems, and prosocial behavior (33). Each subscale score ranged from 0 to 10, with higher scores indicating more problems, except for the prosocial behavior subscale, where higher scores reflected fewer problems. To ensure comparability, ASQ: SE and SDQ scores were normalized to have a mean of zero and a standard deviation of one within the child's age in months and within each survey wave.

For both the Child Cognitive Development and Non-Cognitive Development Status, our examiners underwent professional training. The recruited examiners were undergraduate and graduate students with strong learning abilities and a responsible attitude toward the research. They were trained extensively in the necessary testing skills. Most of the testers are in early childhood education-related majors, so as to interact well with children. All of the enumerators are native Chinese speakers and can understand the local accent.

Specifically, members of our team obtained certifications for the Bayley Scales of Infant and Toddler Development, Third Edition (Bayley-III) and the Wechsler Preschool and Primary Scale of Intelligence Fourth Edition (WPPSI-IV), the Mandarin language version. Certified personnel then trained our recruited examiners, which involved thorough explanations of the test content, watching videos of expert examiners training, and simulating actual testing procedures. Only those who successfully passed a final assessment were allowed to conduct evaluations. Both cognitive assessments were play-based to engage children and provide accurate measurements of their developmental levels. Both assessments were administered one-on-one in the household using a set of standardized toy kit and a detailed record form. The primary caregiver was required to present for holding or supporting the child during test administration, and, when needed, for soothing or reassuring the child and helping the enumerator to communicate test instructions to the child.

For the Non-Cognitive Development status, we utilized the Ages and Stages Questionnaire: Social-Emotional (ASQ-SE) and the Strengths and Difficulties Questionnaire (SDQ). These assessments were completed by primary caregivers of the children. To ensure accuracy, interviewers read the questions aloud to the caregivers, guiding them through the recall process and ensuring that they reported accurately on the child's non-cognitive behaviors.

Addition, we conducted a pilot survey following the training to ensure consistency in the understanding and application of the assessment tools among all examiners.

#### 2.2.4 Child health status

Four key health indicators were collected: height, weight, illness episodes in the past 2 weeks, and hemoglobin concentrations.

Height and weight were measured using standard instruments, and growth indicators (height-for-age *z*-scores, weight-for-age *z*-scores, weight-for-height *z*-scores, and body mass index-for-age *z*-scores) were calculated according to World Health Organization (WHO) standards (34).

Caregivers reported the number of illness episodes occurring in the 2 weeks preceding each survey. To enhance the accuracy of their recall, we implemented a comprehensive training program for our interviewers, equipping them with strategies to facilitate detailed recollections of each illness episode. For instance, if a caregiver noted that their child experienced two colds in the past 2 weeks, the interviewer would probe further, inquiring whether medical care was sought or medication purchased without a healthcare visit. We systematically documented the specific questions posed, which included: “In the past 2 weeks, how many times did your child exhibit symptoms of illness, such as a cold or cough?” and “For each episode, did you seek medical attention, or purchase medication without a healthcare provider?” Additionally, we inquired about the caregiver's expenditures, asking, “How much did the infant or toddler spend on medical care? What was the most serious illness the infant had? How much did the infant or toddler pay for the most serious illness?” By encouraging caregivers to reflect on these specific events and actions, we aimed to significantly improve the reliability of the reported data.

Hemoglobin level was measured by qualified nursing personnel. Anemia in children aged 6 to 23 months is defined as a hemoglobin concentration of <105 g/L. For children aged 24 to 59 months, the threshold is set at a hemoglobin concentration of <110 g/L. In children aged 60 months or older, anemia is indicated by a hemoglobin concentration of <115 g/L (35).

#### 2.2.5 Dietary diversity score

The Dietary Diversity Score (DDS) was based on caregivers' 24-hour recall of the child's dietary intake, following guidelines from WHO and United Nations Children's Fund (UNICEF), and FAO (7, 36). Trained enumerators conducted face-to-face interviews with caregivers using the multiple-pass method to enhance recall accuracy. This method involved three steps: first, caregiver provided an unprompted list of all foods and drinks the child consumed in past 24 h; second, the enumerators probed for specific details regarding food types, portion sizes, and preparation methods to obtain more accurate data; third, a review was conducted to ensure no food items were missed or inaccurately recorded. This structured approach enabled us to gather reliable data on the diversity of the child's diet.

Children aged 6–23 months were assessed using eight food groups, with the minimum dietary diversity requirement met if they consumed at least five food groups (Table 2, Column 1). For children aged 24 months and older, nine food groups were utilized. A DDS below 5 was considered low, while a score of 5 or higher was adequate (19, 37) (Table 2, Column 2). In summary, DDS was categorized into two groups: low (0–4) and adequate (5 and above). We standardized these DDS values to have a mean of zero and a standard deviation of one within the child's age in months and within each wave, ensuring a rigorous normalization process.

**Table 2:** Definition, food groups, and categorization of dietary diversity score by age.

**6–23 months**	**24 months and above**
(1) Breast milk	(1) Starchy staples
(2) Grains, roots, and tubers	(2) Dark-green leafy vegetables
(3) Legumes and nuts	(3) Other vitamin A-rich fruits and vegetables
(4) Dairy products (milk, yogurt, cheese)	(4) Other fruits and vegetables
(5) Flesh foods (including fish, poultry, and liver/organ meats)	(5) Organ meat
(6) Eggs	(6) Meat and fish
(7) Vitamin A-rich fruits and vegetables	(7) Eggs
(8) Other fruits and vegetables	(8) Milk and milk products
	(9) Legumes, nuts, and seeds

### 2.3 Statistical analysis

All statistical analyses were performed using Stata software, version 17.0. We employed one-way analysis of variance (ANOVA) to determine whether there were statistically significant differences in child, household, health, and development characteristics based on low and adequate DDS. Categorical variables are presented as proportions; continuous variables as means and standard deviation (Means ± SD).

For the main analysis, we use Ordinary Least Squares (OLS) regression to estimate the impact of dietary diversity on early childhood development. To address potential endogeneity concerns, such as omitted variable bias or sample selection bias, we incorporated child fixed-effects specification and accounted for clustering effects at the village level in all model settings. We assessed the robustness of the findings using four models, detailed as follows:


(1) $$\begin{array}{lll} \text{Outcom}{\text{e}}_{\text{it}} & = & \text{α}+{\text{β}}_{1}\text{DD}{\text{S}}_{it}+\beta_{2}{\text{X}}_{\text{it}}+{\text{s}}_{\text{it}}+\varepsilon_{\text{it}} \end{array}$$

(2) $$\begin{array}{lll} \text{Outcom}{\text{e}}_{\text{it}} & = & \text{α}+{\text{β}}_{1}\text{DD}{\text{S}}_{\text{it}}+{\text{w}}_{i}+{\text{s}}_{\text{it}}+\varepsilon_{\text{it}} \end{array}$$

(3) $$\begin{array}{lll} \text{Outcom}{\text{e}}_{\text{it}} & = & \text{α}+{\text{β}}_{1}\text{DD}{\text{S}}_{\text{it}}+\beta_{2}{\text{X}}_{\text{it}}+{\text{w}}_{i}+{\text{s}}_{\text{it}}+\varepsilon_{\text{it}} \end{array}$$

(4) $$\begin{array}{l} {\text{Outcome}}_{\text{it}}=\alpha \text{+}\beta_{\text{1}}{\text{DDS}}_{\text{it}}\text{+}\beta_{\text{2}}{\text{X}}_{\text{it}}\text{+ Outcome}\_{\text{lag}}_{\text{i}}\\ \;+w_{i}+s_{it}+\varepsilon_{it} \end{array}$$

(5) $$\begin{array}{lll} \text{Outcom}{\text{e}}_{\text{it}} & = & \text{α}+{\text{β}}_{1}\text{DD}{\text{S}}_{\text{it}}+\beta_{2}{\text{X}}_{\text{it}}+\eta_{i}+{\text{w}}_{i}+{\text{s}}_{\text{it}}+\varepsilon_{\text{it}} \end{array}$$

Where i and t represent the child and the survey period, where *Outcome*_*it*_ is dependent variables for the child, including children's cognition, verbal comprehension, difficulties score, prosocial behavior score, anemia, times ill in past 2 weeks, height-for-age z-scores, weight-for-age z-scores, weight-for-height z-scores, and body mass index-for-age z-scores; *DDS*_*it*_ denotes main independent variables, which has two forms, a continuous variable ranging from 0 to 9, and a categorical variable indicates two DDS groups: low and adequate DDS, we choose low DDS as the omitted reference category; The coefficient of interest is β_1_, is interpreted as the effect of consuming diverse diets. *w*_*i*_ are survey wave indicators, *s*_*it*_ are fixed effect for enumerators designed to capture measurement error; ε_*it*_ is an error term. Standard errors were clustered at the village level to account for correlation within villages as well as serial correlation over time.

Table 4 uses Model 1, which includes control variables *X*_*it*_, enumerator fixed effects ε_*it*_, and error term ε_*it*_. Models 2–5 (presented in Tables 5, 6) incrementally incorporate child and household-level control variables, lagged terms for the dependent variables (*Outcom*_*e*_*l*_*agi*_), and child fixed effects (η_*i*_).

All statistical analyses were conducted using Stata software version 17.0. Statistical significance was considered at *p* < 0.05, with standard errors adjusted for clustering at the village level.

## 3 Results

### 3.1 Variation of dietary diversity score by age of children

Table 1 presents a comprehensive overview of children's dietary diversity score across the four survey waves, showing a consistent upward trend over time. At 6–23 months of age, only approximately a quarter of children (24%) meet the minimum dietary diversity criteria, with a mean DDS of 3.38. The prevalence of adequate DDS increased significantly as the infants grew older. Notably, at 51–83 months (wave 3), 70% of surveyed children are classified as having adequate dietary diversity, with a mean DDS of 5.34, which is associated with an increased probability of adequate micronutrient intake. This represents a marked increase of about 58% in mean DDS from ages 6–23 to 51–83 months. The variations in both the mean DDS and the proportions of the DDS categories across different age groups indicate a discernible improvement in children's diet quality with advancing age (38).

### 3.2 Sample characteristics and development outcomes by DDS category

We present descriptive statistics to characterize DDS during early childhood, comparing the characteristics of children and households with low and adequate DDS (Table 3).

**Table 3:** Sample characteristics and development outcomes by low and adequate dietary diversity score in the baseline survey.

**Characteristics**	**Full sample**	**Low DDS**	**Adequate DDS**	***P*-value**
**Child characteristics**				
Dietary diversity score	3.38 ± 1.58	2.72 ± 1.15	5.44 ± 0.66	<0.001
Age of child (months)	12.87 ± 4.86	12.48 ± 4.92	14.11 ± 4.46	<0.001
Male (1 = yes, 0 = no)	52.0	53.3	48.0	0.11
Premature birth (1 = yes, 0 = No)	4.6	4.7	4.4	0.84
Mode of delivery (1 = natural, 0 = otherwise)	75.4	74.8	77.2	0.41
Ever-breastfeeding (1 = yes, 0 = no)	91.6	90.3	95.9	0.002
Having siblings (1 = yes, 0 = no)	34.9	34.9	34.7	0.94
Observations	1,207	913	294	
**Household characteristics**				
Grandmother's health status (1 = health)	26.1	24.1	32.3	0.005
Mother is the primary caregiver (1 = yes, 0 = no)	79.7	77.5	86.4	0.001
Primary caregiver's education (1 = high school or above)	16.4	14.7	21.8	0.004
Age of mother above 27 (1 = yes, 0 = no)	50.4	50.5	50.0	0.89
Father's education (1 = high school or above)	17.4	17.0	18.7	0.50
Asset index	−1.25 ± 1.33	−1.36 ± 1.34	−0.91 ± 1.25	<0.001
Government welfare recipient (1 = yes, 0 = no)	11.4	10.8	13.3	0.26
Observations	1,207	913	294	
**Child development**				
**Child cognition and non-cognition**				
Cognitive score	95.28 ± 12.70	94.63 ± 12.82	97.29 ± 12.11	0.002
Cognition delay (1 = yes, 0 = no)	56.7	58.5	51.2	0.03
Language score	91.63 ± 13.51	90.80 ± 13.31	94.22 ± 13.81	<0.001
Language delay (1 = yes, 0 = no)	64.1	66.5	56.7	0.002
Observations	1,206	913	293	
Social-Emotional delay (1 = yes, 0 = no)	48.0	50.7	38.5	0.003
Observations	830	643	187	
**Child health**				
Anemia (1 = yes, 0 = no)	35.66	34.97	37.86	0.379
Observations	1,158	878	280	
Times of illness in the past 2 weeks	3.45 ± 3.87	3.68 ± 3.96	2.74 ± 3.48	<0.001
Observations	1,207	913	294	
Height for age *z*-scores	0.32 ± 1.26	0.34 ± 1.30	0.25 ± 1.13	0.25
Stunting (1 = yes, 0 = no)	2.70	2.89	2.10	0.47
Observations	1,187	901	286	
Weight for age *z*-scores	0.61 ± 0.98	0.64 ± 1.00	0.53 ± 0.90	0.12
Underweight (1 = yes, 0 = no)	0.58	0.78	0.00	0.13
Observations	1,190	902	288	
Weight for height *z*-scores	0.44 ± 1.06	0.47 ± 1.06	0.37 ± 1.06	0.20
Wasting (1 = yes, 0 = no)	0.94	1.02	0.70	0.63
Observations	1,165	881	284	
BMI for age *z*-scores	0.57 ± 1.10	0.59 ± 1.10	0.51 ± 1.12	0.31
Observations	1,165	881	284	

DDS, dietary diversity score; BMI, body mass index.

Results are given as mean ± standard deviation or as %.

Three notable findings emerge. First, at baseline, a substantial fraction of children in our sample are delayed and malnourished. Specifically, among children aged 6–23 months, 56.7% exhibit cognitive delays, and 64.1% show delays in language development. Additionally, almost 48% of children are at risk of social-emotional delays, and nearly one-third are anemic. These statistics are consistent across both low DDS and adequate DDS groups.

Second, we find positive associations between adequate DDS and favorable child outcomes, as well as characteristics of children and households. Children with high dietary diversity scores tend to have higher cognitive (*p* < 0.01) and language (*p* < 0.001) scores, less frequent illness (*p* < 0.001), and better socio-emotional skills (*p* < 0.01).

Third, children with high dietary diversity scores are more likely to come from wealthier households (*p* < 0.001) and to be cared for by mothers with higher levels of education (*p* < 0.01). Moreover, children who were ever breastfed exhibit greater dietary diversity (*p* < 0.01), and the grandmothers of children with higher DDS tend to be healthier (*p* < 0.01). Notably, no significant differences in DDS categories were found regarding other characteristics of children and households.

### 3.3 Relationship between DDS and childhood development

The primary results regarding the impact of dietary diversity score on childhood development are shown in Table 4. Comprehensive regression analysis reveals a substantial positive correlation between dietary diversity and the cognitive and language development of children aged 6–23 months (wave 0, *p* < 0.01). As children advance to 16–54 months (wave 1–wave 2), the influence of dietary diversity extends beyond cognitive and language skills to positively affect non-cognitive abilities (*p* < 0.001). Particularly noteworthy is the correlation between elevated dietary diversity and superior non-cognitive capabilities among children aged 51–83 months (wave 3, *p* < 0.001).

**Table 4:** The effect of dietary diversity score on children's cognition, non-cognition, and health outcomes over time.

	**Cognitive standard score**	**Language standard score**	**Prosocial behavior standard score**	**Difficulty standard score**	**Height for age *z*-scores**	**Weight for age *z*-scores**	**Weight for age *z*-scores**	**BMI for age *z*-scores**	**Anemia**	**Times of illness in the past 2 weeks**
DDS in wave 0	0.053^**^	0.068^***^	0.053	0.053	0.010	−0.009	−0.015	−0.012	0.007	−0.353^***^
	(0.019)	(0.017)	(0.028)	(0.028)	(0.025)	(0.016)	(0.024)	(0.025)	(0.009)	(0.081)
Observations	1,207	1,207	830	830	1,187	1,190	1,168	1,181	1,207	1,207
DDS in wave 1	0.042^**^	0.044^**^	0.087^***^	0.087^***^	0.027	0.012	−0.001	−0.008	0.001	−0.251^***^
	(0.016)	(0.015)	(0.016)	(0.016)	(0.017)	(0.015)	(0.018)	(0.019)	(0.007)	(0.060)
Observations	1,640	1,636	1,621	1,621	1,615	1,615	1,613	1,612	1,606	1,654
DDS in wave 2	0.051^*^	0.053^*^	0.099^***^	0.099^***^	0.020	−0.002	−0.021	−0.026	−0.015	−0.306^***^
	(0.022)	(0.022)	(0.020)	(0.020)	(0.019)	(0.017)	(0.021)	(0.021)	(0.008)	(0.064)
Observations	1,222	1,221	1,227	1,227	1,163	1,164	1,161	1,160	1,216	1,248
DDS in wave 3	0.029	0.029	0.059^***^	0.063^***^	0.026	0.000	−0.043^*^	−0.020		
	(0.018)	(0.019)	(0.014)	(0.017)	(0.018)	(0.017)	(0.019)	(0.019)		
Observations	1,330	1,330	1,344	1,343	1,281	1,292	1,163	1,276		

DDS, dietary diversity score; BMI, body mass index.

^*^p <0.05.

^**^p <0.01.

^***^p <0.001.

The table shows coefficients, and all standard errors account for clustering at the village level standard errors. All regressions control for examiner fixed effects, and controls include the child's age, gender, premature birth, natural birth, ever-breastfeeding, whether he/she has siblings, grandmother's health status, whether the mother is the primary caregiver, primary caregiver's education level, age of mother, father's education level, asset index, whether the household receives government welfare.

Regarding health outcomes, our findings indicate that heightened dietary diversity is correlated with a reduced frequency of illnesses among children aged 6–54 months, with the association being statistically significant (*p* < 0.001). Furthermore, among children aged 51–83 months (wave 3), increased dietary diversity is significantly associated with a reduction in the incidence of illnesses, as assessed by the World Health Organization (39). Additionally, it is possible that children consuming higher quality diets are at less risk for excess weight gain.

Table 5 presents the results of multivariate analysis to check the robustness of the above findings. The estimated effect of dietary diversity score is shown across different models: Model 1 (Column 1, Pooled OLS) displays estimates from ordinary least squares (OLS) regressions, controlling only for the survey wave and enumerator dummies; Model 2 (Column 2, Pooled OLS With Controls) adds additional time-variant control variables (child and household covariates); Model 3 (Column 3, LDV) reports the Lagged Dependent Variable (LDV) Model which additionally accounts for lagged outcomes. The LDV model can address autocorrelation issues, specifically the correlation between the dependent variable and its lagged value. Finally, Model 4 (Column 4, Fixed Effects) incorporates child fixed effects, time-variant control variables, as well as wave and enumerator dummies.

**Table 5:** Effect of dietary diversity score on children's cognition, non-cognition, and health outcomes.

	**Pooled OLS**	**Pooled OLS with controls**	**LDV**	**Fixed effects**
	**(1)**	**(2)**	**(3)**	**(4)**
**Child cognition**				
**Cognitive standard score**				
Dietary diversity score	0.107^***^	0.069^***^	0.047^*^	0.011
	(0.016)	(0.016)	(0.022)	(0.015)
Observations	5,388	5,388	2,598	5,388
**Language standard score**				
Dietary diversity score	0.118^***^	0.073^***^	0.034	−0.002
	(0.015)	(0.015)	(0.020)	(0.016)
Observations	5,388	5,388	2,598	5,388
**Child non-cognition**				
**Prosocial behavior standard score**				
Dietary diversity score	0.145^***^	0.124^***^	0.099^***^	0.066^**^
	(0.017)	(0.017)	(0.021)	(0.022)
Observations	5,021	5,021	2,493	5,021
**Difficulty standard score**				
Dietary diversity score	0.154^***^	0.127^***^	0.099^***^	0.068^**^
	(0.018)	(0.017)	(0.019)	(0.021)
Observations	5,021	5,021	2,493	5,021
**Child health**				
**Anemia**				
Dietary diversity score	−0.010	−0.008	−0.035^**^	−0.016
	(0.007)	(0.008)	(0.013)	(0.010)
Observations	3,739	3,739	1,353	3,739
**Times of illness in the past 2 weeks**				
Dietary diversity score	−0.426^***^	−0.486^***^	−0.445^***^	−0.522^***^
	(0.055)	(0.059)	(0.100)	(0.102)
Observations	4,109	4,109	1,572	4,109
**Height for age z-scores**				
Dietary diversity score	0.048^**^	0.030	0.033^*^	0.019
	(0.017)	(0.017)	(0.014)	(0.016)
Observations	5,246	5,246	2,470	5,246
**Weight for age z-scores**				
Dietary diversity score	0.016	0.001	−0.007	−0.004
	(0.014)	(0.013)	(0.013)	(0.012)
Observations	5,261	5,261	2,490	5,261
**Weight for height z-scores**				
Dietary diversity score	−0.021	−0.030	−0.046^*^	−0.031
	(0.016)	(0.016)	(0.019)	(0.016)
Observations	5,100	5,100	2,372	5,100
**BMI for age z-scores**				
Dietary diversity score	−0.023	−0.031	−0.047^*^	−0.031
	(0.017)	(0.017)	(0.019)	(0.017)
Observations	5,100	5,100	2,372	5,100
Characteristics of child and household	No	Yes	Yes	Yes
Examiner fixed effects	Yes	Yes	Yes	Yes
Wave fixed effects	Yes	Yes	Yes	Yes
Lagged outcomes	No	No	Yes	No
Child fixed effects	No	No	No	Yes

BMI, body mass index.

^*^p <0.05.

^**^p <0.01.

^***^p <0.001.

The table shows coefficients and standard errors, and all standard errors account for clustering at the village level standard errors. Column 1 displays estimates from ordinary least squares (OLS) regressions controlling only for the survey wave and tester fixed effects. Column 2 shows coefficients from pooled OLS regressions additionally controlling for child and household covariates including the child's age, gender, premature birth, natural birth, ever-breastfeeding, whether he/she has siblings, grandmother's health status, whether the mother is the primary caregiver, primary caregiver's education level, age of mother, father's education level, asset index, whether the household receives government welfare. Column 3 reports the Lagged Dependent Variable Model (LDV), which incorporates the previous period's various developmental scores. Column 4 is a two-way fixed effects model, which includes controls for child and household characteristics, as well as wave, tester, and child fixed effects.

In the first column of Pooled OLS, we observe a positive and significant association between child dietary diversity and various aspects of child development, including cognitive score, language score, prosocial behavior, difficulty score, height for age *z*-score, and times of illness in the past 2 weeks. In Column 2, after controlling for additional child and household characteristics, similar results are maintained, although the height-for-age *z*-score is no longer significantly correlated with DDS. When further controlling for lagged child development variables in Column 3, most positive correlations persist; however, the coefficient for the language standard score becomes insignificant, while the coefficient on height for age *z*-score becomes positively significant again. Conversely, the estimated effect of dietary diversity score on anemia, weight for height *z*-scores, and body mass index for age *z*-scores becomes significantly negative.

Our main estimates using two-way fixed effects are presented in Column 4, where the associations remain statistically significant after adjusting for time-invariant heterogeneity. Specifically, DDS is associated with a 0.066-point increase in prosocial behavior score (*P* < 0.01), a 0.068-point increase in difficulty score (*P* < 0.01), and about 7-days decrease in times of illness in the past 2 weeks (*P* < 0.001). When regarding DDS as a binary categorical variable, consistent results are observed, as shown in Table 6.

**Table 6:** Robustness test of the effect of low and adequate dietary diversity score on children's cognition, non-cognition, and health outcomes.

	**Pooled OLS**	**Pooled OLS with controls**	**LDV**	**Fixed effects**
	**(1)**	**(2)**	**(3)**	**(4)**
**Child cognition**				
**Cognitive standard score**				
Low DDS	Ref	Ref	Ref	Ref
Adequate DDS	0.194^***^	0.134^***^	0.109^**^	0.018
	(0.033)	(0.033)	(0.040)	(0.028)
Observations	5,388	5,388	2,598	5,388
**Language standard score**				
Low DDS	Ref	Ref	Ref	Ref
Adequate DDS	0.211^***^	0.139^***^	0.074	0.000
	(0.031)	(0.030)	(0.041)	(0.032)
Observations	5,388	5,388	2,598	5,388
**Child non-cognition**				
**Prosocial behavior standard score**				
Low DDS	Ref	Ref	Ref	Ref
Adequate DDS	0.281^***^	0.246^***^	0.241^***^	0.152^***^
	(0.037)	(0.035)	(0.043)	(0.042)
Observations	5,021	5,021	2,493	5,021
**Difficulty standard score**				
Low DDS	Ref	Ref	Ref	Ref
Adequate DDS	0.260^***^	0.216^***^	0.163^***^	0.109^*^
	(0.038)	(0.036)	(0.042)	(0.042)
Observations	5,021	5,021	2,493	5,021
**Child health**				
**Anemia**				
Low DDS	Ref	Ref	Ref	Ref
Adequate DDS	−0.008	−0.001	−0.065^*^	0.015
	(0.015)	(0.015)	(0.027)	(0.024)
Observations	3,739	3,739	1,353	3,739
**Times of illness in the past 2 weeks**				
Low DDS	Ref	Ref	Ref	Ref
Adequate DDS	−0.609^***^	−0.682^***^	−0.660^***^	−0.657^***^
	(0.116)	(0.125)	(0.189)	(0.185)
Observations	4,109	4,109	1,572	4,109
**Height for age z-score**				
Low DDS	Ref	Ref	Ref	Ref
Adequate DDS	0.050	0.033	0.044	0.008
	(0.034)	(0.034)	(0.030)	(0.031)
Observations	5,246	5,246	2,470	5,246
**Weight for age z-score**				
Low DDS	Ref	Ref	Ref	Ref
Adequate DDS	0.001	−0.010	−0.022	−0.014
	(0.029)	(0.027)	(0.025)	(0.022)
Observations	5,261	5,261	2,490	5,261
**Weight for height z-score**				
Low DDS	Ref	Ref	Ref	Ref
Adequate DDS	−0.052	−0.055	−0.064	−0.040
	(0.034)	(0.034)	(0.036)	(0.034)
Observations	5,100	5,100	2,372	5,100
**BMI for age z-score**				
Low DDS	Ref	Ref	Ref	Ref
Adequate DDS	−0.053	−0.056	−0.065	−0.038
	(0.036)	(0.036)	(0.037)	(0.036)
Observations	5,100	5,100	2,372	5,100
Characteristics of child and household	No	Yes	Yes	Yes
Examiner fixed effects	Yes	Yes	Yes	Yes
Wave fixed effects	Yes	Yes	Yes	Yes
Lagged outcomes	No	No	Yes	No
Child fixed effects	No	No	No	Yes

DDS, dietary diversity score; BMI, body mass index.

^*^p <0.05.

^**^p <0.01.

^***^p <0.001.

The table shows coefficients and standard errors, and all standard errors account for clustering at the village level standard errors. Column 1 displays estimates from ordinary least squares (OLS) regressions controlling only for the survey wave and tester fixed effects. Column 2 shows coefficients from pooled OLS regressions additionally controlling for child and household covariates including the child's age, gender, premature birth, natural birth, ever-breastfeeding, whether he/she has siblings, grandmother's health status, whether the mother is the primary caregiver, primary caregiver's education level, age of mother, father's education level, asset index, whether the household receives government welfare. Column 3 reports the Lagged Dependent Variable Model (LDV), which incorporates the previous period's various developmental scores. Column 4 is a two-way fixed effects model, which includes controls for child and household characteristics, as well as wave, tester, and child fixed effects.

## 4 Discussion

Appropriate feeding practices ensure the adequate provision of nutrients from the early stages of life, which are crucial for healthy physical and mental development, as well as long-term health. Despite the significant impact of dietary diversity on the development of children and adult health, a substantial portion of children (69%) fail to meet dietary diversity standards (10). Utilizing panel data from 1,207 infants and their families across four waves of surveys, this study investigates the impact of dietary diversity on mitigating poor infant health, malnutrition, and early childhood development challenges in rural China, a representative low- and middle-income country.

The study found that the majority of children did not meet the dietary diversity standard before the age of two. However, as children grew older, there was a significant improvement in dietary diversity. This finding aligns with results from other low- and middle-income countries, where 75% children failed to achieve dietary diversity before the age of two (17, 20, 40). Moreover, several studies conducted among children and adolescents indicate higher proportions achieving dietary diversity. For instance, 31% of 1,066 children aged 24–59 months in rural Burkina Faso (41), 85% of 3–5-year-old preschoolers in rural China (19), 57% of 5- to 12-year-old schoolchildren in South Africa (42), and 58% of 365 high school adolescents in Ethiopia (43) exhibited high DDS. This improvement in older children may be attributed to various factors, including the introduction of complementary foods, a shift toward consuming family diet, increased opportunities to access a wider variety of foods, and improved appetites (12).

Although dietary diversity generally increases with age, notable disparities exist among families. We found that infants whose primary caregivers and mothers with more than 9 years of education exhibit higher DDS. These results are consistent with many studies linking parental education levels to elevated DDS (13, 44). The dietary quality of children during their early years primarily depends on the behaviors and decisions of their primary caregiver. One possible explanation is that higher education enhances awareness of healthy dietary practices through improvements in health knowledge, literacy, wealth, and prenatal care utilization (45). Furthermore, mothers or grandmothers with good health could contribute to higher DDS among the children in our sample. And household financial resources play a pivotal role in determining the affordability and accessibility of food, thereby shaping dietary patterns (46, 47).

We also found that DDS is significantly associated with infant health and nutrition in rural China. The research revealed that dietary diversity effectively reduces the frequency of illnesses in children across all age groups, yielding consistent and robust outcomes. Using in-depth panel data allows for the longitudinally tracking of infants and addresses endogeneity concerns through child fixed effects. Similar conclusions have been reported in other studies surveyed in rural China (22), where higher dietary diversity is linked to elevated micronutrient intake among children (41, 48), consequently reducing illness frequency (49, 50).

Our findings revealed that dietary diversity contributes to the enhancement of non-cognitive abilities in children aged over the age of 2 years. Nevertheless, while dietary diversity effectively promotes cognitive abilities in children aged 6–54 months, this effect does not extend to those aged 51–83 months. This discrepancy might be attributed to the rapid development of cognitive abilities during early stages, where ample stimuli can effectively foster children's cognitive growth (51, 52). In contrast, in older children, brain development progresses at a slower pace, leading to reduced nutritional requirements to support cognitive functions such as working memory and inhibition (53, 54).

Moreover, the development of non-cognitive abilities is closely linked to early cognitive capabilities. Our findings suggest that dietary diversity plays a vital role in enhancing non-cognitive abilities in later stages of childhood. This aligns with conclusions from other studies. For instance, research among preschoolers in rural China established an association between healthy dietary diversity and a decreased likelihood of presenting symptoms related to hyperactivity/inattention, peer relationship problems, and prosocial behavior (55, 56). Furthermore, higher dietary quality or diversity has been correlated with improved non-cognition functions in adolescents, adults, and older individuals (57–59). These findings highlight the importance of promoting dietary diversity across all age groups.

Our study has four limitations that should be addressed in future research. First, as our survey is limited to a rural region in northwestern China, we cannot claim that our results are representative of rural China as a whole. Given the potential variations in the utilization of dietary diversity and its correlations with infant health, nutrition, and development outcomes across different regions, further studies in other areas of China and other low- and middle-income countries are recommended. Second, many factors related to early childhood development extend beyond infant feeding. Essential considerations include interactive parenting inputs, the availability of play materials and books at home, early childhood education, and attendance at kindergarten and primary school. Future studies should analyze the relative importance of parenting practices and nutrition across different age groups to delineate their combined effects on health, nutrition, and early childhood development. By integrating these factors, researchers can gain a more comprehensive understanding of the determinants of cognitive outcomes in children, leading to more effective interventions and policies tailored to specific developmental stages. Third, our study utilized only one dietary diversity assessment tool, and we alternative tools might yield different results. Therefore, incorporating additional assessment methods in future research is recommended to ensure comprehensive and comparable findings. Lastly, while we acknowledge the importance and relevance of heterogeneity and interaction analyses, we opted not to perform extensive interaction analyses in this study due to the limited sample size and our primary research objectives. However, we fully recognize the value of exploring potential subgroup effects and suggest that future research with larger and more diverse samples examine these heterogeneity effects to further elucidate the complex relationship between dietary diversity and child development.

## 5 Conclusion

Early nutrition plays a crucial role in shaping both childhood and long-term developmental outcomes. Our findings reveal that most children under the age of two fail to meet the recommended dietary diversity standards, though there is some improvement beyond this age. Additionally, our study underscores the significant influence of socio-economic factors on DDS, with wealthier households exhibiting higher DDS. Ever-breastfeeding also emerged as a key determinant of higher DDS, showing a positive correlation.

Moreover, the impact of dietary diversity on child development is both immediate and sustained, influencing cognitive abilities in the short term and non-cognitive skill over time. While higher DDS is associated with better cognitive and non-cognitive outcomes and reduced illness incidence, the strength of these relationships varies. Specifically, DDS demonstrates a strong initial correlation with cognitive development outcomes, but this link diminishes as children reach preschool age. Conversely, the positive association between DDS and non-cognitive development intensifies after age two, indicating a longer-lasting effect in this domain.

## 6 Implications for research and practice

Based on the study's findings, it is crucial for policymakers to adopt a more strategic approach to improving child development and feeding practices in rural areas. Beyond merely raising awareness, targeted interventions should be implemented, such as the establishment of community-based programs that provide hands-on training and support for caregivers. These programs could involve local healthcare workers, educators, and community leaders in delivering accessible and culturally relevant guidance on effective child-rearing and feeding practices. Additionally, integrating nutritional support into existing healthcare and social services would ensure that families receive both the knowledge and resources necessary to improve children's health, cognitive, and non-cognitive outcomes. By embedding these practices into a broader policy framework and allocating appropriate resources, long-term, sustainable improvements in early childhood development can be achieved.

## Data Availability

The original contributions presented in the study are included in the article/supplementary material, further inquiries can be directed to the corresponding author.
